# Dimensions of Velopharyngeal Space following Maxillary Advancement with Le Fort I Osteotomy Compared to Zisser Segmental Osteotomy: A Cephalometric Study

**DOI:** 10.1155/2015/389605

**Published:** 2015-07-26

**Authors:** Furkan Erol Karabekmez, Johannes Kleinheinz, Susanne Jung

**Affiliations:** ^1^Department of Plastic, Reconstructive and Aesthetic Surgery, Faculty of Medicine, Abant Izzet Baysal University, Golkoy, 14280 Bolu, Turkey; ^2^Oral and Maxillofacial Surgery Clinics, Central Hospital, University Hospital Munster, Albert Schweitzer Campus 1, 48149 Münster, Germany

## Abstract

The objectives of this study are to assess the velopharyngeal dimensions using cephalometric variables of the nasopharynx and oropharynx as well as to compare the Le Fort I osteotomy technique to Zisser's anterior maxillary osteotomy technique based on patients' outcomes within early and late postoperative follow-ups. 15 patients with severe maxillary deficiency treated with Le Fort I osteotomy and maxillary segmental osteotomy were assessed. Preoperative, early postoperative, and late postoperative follow-up lateral cephalograms, patient histories, and operative reports are reviewed with a focus on defined cephalometric landmarks for assessing velopharyngeal space dimension and maxillary movement (measured for three different tracing points). A significant change was found between preoperative and postoperative lateral cephalometric measurements regarding the distance between the posterior nasal spine and the posterior pharyngeal wall in Le Fort I osteotomy cases. However, no significant difference was found between preoperative and postoperative measurements in maxillary segmental osteotomy cases regarding the same measurements. The velopharyngeal area calculated for the Le Fort I osteotomy group showed a significant difference between the preoperative and postoperative measurements. Le Fort I osteotomy for advancement of upper jaw increases velopharyngeal space. On the other hand, Zisser's anterior maxillary segmental osteotomy does not alter the dimension of the velopharyngeal space significantly.

## 1. Introduction

Patients with cleft lip and palate have changes of the maxilla concerning anatomical dimension, position, and function with diverse prevalence of these changes, which are caused by genetic, developmental, and treatment-associated determinants [1, 2]. In planning secondary orthognathic surgery, the jaw and occlusal relations must be considered in addition to the functional aspects of the pathology [3, 4]. 

*(1) Velopharyngeal Function.* Length and position of the velum may lead to speech impairment or borderline compensated speech [5].

*(2) Reduced Maxillary Length (Shortened Maxilla).* It lacks space in the dentate area for prosthodontic treatment (bridges, implants) [6].

*(3) Maxillary Retrognathia.* It may lead to esthetical and functional complaints [7].

*(4) Velopharyngeal Flap (Velopharyngoplasty).* It, completed at an early stage of growth, may result in a reduced anterior growth of the maxilla [8].

Frequency of maxillary osteotomy for advancement is correlated with the spectrum of severity of labiopalatal clefting [9]. Advancement of the maxilla by a Le Fort I osteotomy carries the risk of increased velopharyngeal space and deterioration of speech function, ending up in hypernasal resonance [10–12], advancement of a short maxilla without gaining new space for prosthodontic treatment, and prevention of sufficient anterior movement caused by the velopharyngeal flap.

Maxillary segmental osteotomy, first described by Zisser (1969) [13, 14], advances the anterior maxillary segment without disturbing nasopharyngeal function. Several modifications of segmental osteotomy such as transpalatal or segmental osteotomy have been described for the maxilla [15, 16]. It seems that using a segmental osteotomy may prevent foreseen problems of conventional maxillary osteotomy for advancement such as disturbance of nasopharyngeal function. However, it is important to define specific indications for when each treatment should be utilized.

In this study, a cephalometric analysis was conducted to evaluate and compare the position of the maxilla, the length of the maxilla, and dimension of the velopharyngeal space after Le Fort I and segmental osteotomy.

## 2. Patients and Methods

After having an approval from local ethics committee in 2013, patient charts were reviewed with the key words “maxillary osteotomy” between 1997 and 2012 in the surgical records of one surgeon in order to make comparison between different surgical procedures without any effect of surgeon's personal preferences. After exclusion of syndromic cases, patients who had maxillary osteotomy previously and had additional systemic disorders, 38 maxillary osteotomy patients were identified initially. Subsequently, 15 were excluded due to lack of records, four patients were excluded due to a setback procedure, and five patients were excluded due to less than 4 mm of advancement or multiple pieces of osteotomy. Therefore, seven patients who had the Zisser operation (named the Zisser group) and seven patients who underwent Le Fort I osteotomy (named the Le Fort group) were included in the study. Patient's characteristics and lateral cephalograms were recorded digitally and numbered by a different author (Susanne Jung) to deidentify cephalograms and ensure blinded measurements by the observer (another author, Furkan Erol Karabekmez). Preoperative cephalograms, taken at the closest date to the surgical procedure, were used for preoperative evaluation and were grouped as T1. Early postoperative cephalograms taken at closest date to the surgical procedure were named T2. The late postoperative cephalograms taken on the latest follow-up of the patient were named T3. Complications and date of the removal of elastics were also recorded.

### 2.1. Cephalometric Evaluation

**Figure 1:**
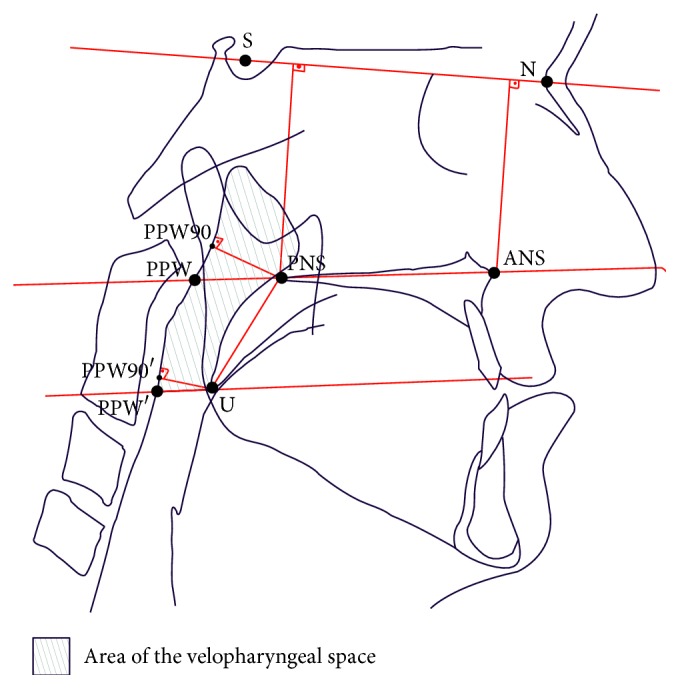
Variables used for cephalometric evaluations:* points*: ANS, most anterior point of anterior nasal spine; PNS, posterior nasal spine; PPW, posterior pharyngeal wall; U, tip of the uvula; S, midpoint of hypophyseal fossa; N, most anterior point of frontonasal suture.* Planes*: SN, sella-nasion plane; PP, palatal plane.* Distances*: PNS-PPW, distance measured parallel to the PP from PNS to PPW; PNS-PPW90, distance measured with line drawn perpendicular from PNS to PPW90; U-PPW′, distance measured parallel to the PP from U to PPW′; U-PPW90′, distance measured with line drawn perpendicular from U to PPW90′; ANS-PNS, distance measured ANS to PNS (for the maxillary dental arch length); PNS-U, distance measured from PNS to U; ANS-SN, distance measured with line drawn perpendicular from ANS to SN; PNS-SN, distance measured with line drawn perpendicular from PNS to SN.* Angle*: SN-PP, angle between SN and PP.* Area*: area of the airway located above the line drawn parallel to the PP and passing through U point.

The standard lateral cephalometric radiographs were transferred to digital images using a digital camera (DSC-W90, Sony Corp., Tokyo, Japan). The landmarks on the lateral cephalometric radiographic images were traced using Image J software (National Institutes of Health, http://rsbweb.nih.gov/, USA). After calibration with the scale, the following were measured using the “measure” tools of the program: posterior nasal spine (PNS) to posterior pharyngeal wall (PPW) distance parallel to the palatal plane (PP), PNS to PPW distance perpendicular to the PPW, tip of the uvula (U) to PPW distance parallel to the PP, U to PPW distance perpendicular to the PPW, anterior nasal spine (ANS) to PNS distance (for the maxillary dental arch length), PNS to U distance, sella-nasion plane (SN) PP angle (SN-PP), ANS to SN distance, and PNS to SN. The area located superior to the U-PPW line parallel to the PP was calculated. Points, distances, and areas used in the study are showed in Figure 1. Each measurement was calculated for T1, T2, and T3 time points by the same blinded observer. Different author performed the cephalometric tracings and the surgery.

### 2.2. Surgery

For Le Fort I surgery, patients are induced with nasotracheal hypotensive general anaesthesia and prepared for a standard intraoral Le Fort I procedure. Rigid skeletal fixation using a couple of 2.0 mm miniplates and screws to both sides was performed in all patients.

For surgery with the Zisser technique, presurgical orthodontic treatment includes the preparation of space for vertical interdental osteotomies between the second premolar and first molar on the upper jaw (between 15-16 and 25-26, according to the Palmer Notation Method). Surgery was performed following the standard procedure.

**Figure 2:**
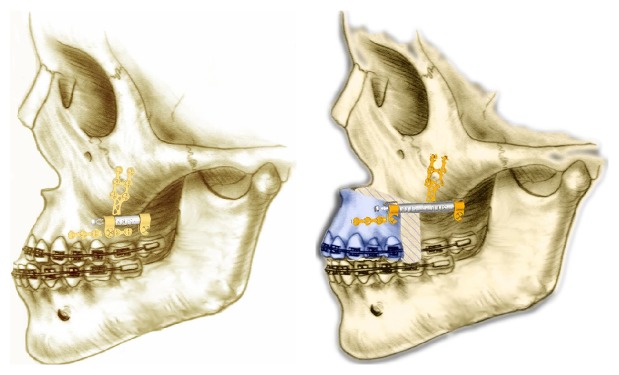
Vertical interdental osteotomies between second premolar and first molar on upper jaw according to Zisser technique followed by distractor application and activation.

In the case of the distractor application, after the Le Fort I or segmental osteotomy, fixation was performed by using a bone born distractor (Medartis, Modus MDO 2.0, Basel, Switzerland) (Figure 2). The maxilla was advanced at a rate of 0.5 mm twice a day after a five-day latency period. The vector of the distractor was planned according to the vertical deficiency, if it existed. The amount of distraction was also determined according to the need of each patient.

### 2.3. Statistical Analysis

The angular, linear, and area measurements were compared using a Wilcoxon signed ranks test to assess the changes between T1 and T2 (as the surgical change); T2 and T3 (as the postsurgical change); and T1 and T3 in both Le Fort I and Zisser groups. Differences between surgical and postsurgical movements of the Le Fort I and Zisser groups' measurements were also compared with Mann-Whitney tests. Distractions versus nondistraction and cleft versus noncleft comparisons were also completed with Mann-Whitney tests.

Correlations between cleft palate history, distraction, and the measured parameters were investigated with the Spearman correlation coefficient test.

The intraobserver reliability was tested with the intraclass correlation coefficient test. Measurements were repeated 1 month later by the same observer. Statistical analyses were performed with PASW (version 18) software (SPSS Inc., Chicago, IL). The results are shown as the mean and the standard deviation; *P* < 0.05 was considered as significant.

## 3. Results

Mean follow-up time for the late postoperative lateral cephalogram (T3) was 20.5 months. T1 cephalograms were obtained mean 28.3 days preoperatively. T2 cephalograms were obtained mean 19.5 days postoperatively. Of the 14 patients, 8 were females and 6 were males. The age at the time of surgery was 15–36 years (mean 21 years). Six of the seven patients in the Zisser group and three of the seven patients in the Le Fort group have cleft lip palate. Four of the seven patients in the Le Fort group and three of the seven in the Zisser group had distraction osteogenesis. Patients' characteristics were summarized in Table 1. Variables used for cephalometric evaluations, including velopharyngeal soft tissue points, are shown in Figure 1.

**Figure 3:**
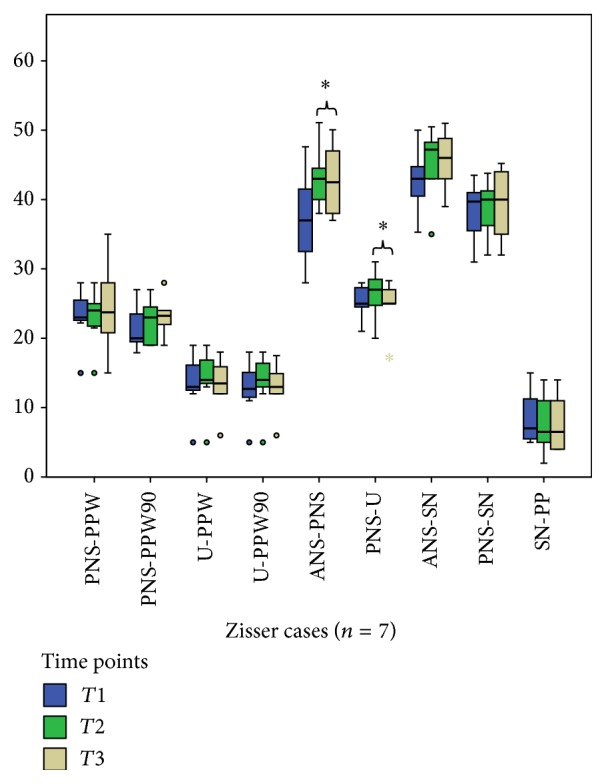
A statistically significant difference found between preoperative (T1) and postoperative (T2) ANS-PNS distances and postoperative (T2) and late postoperative (T3) PNS-U distances in the Zisser group. (Box plots show the median, interquartile range, 95% percentile, and outliers as circles. *∗* indicates significant difference.)

**Figure 4:**
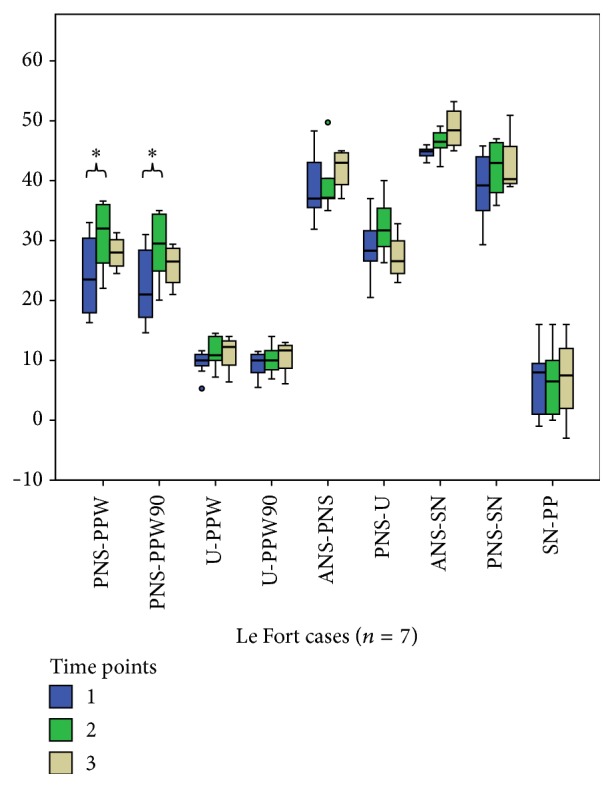
A statistically significant difference was found between T1 and T2 measurements for PNS-PPW and PNS-PPW90 in the Le Fort group. (Box plots show the median, interquartile range, 95% percentile, and outliers as circles. *∗* indicates significant difference.)

Comparisons between T1 and T2 within the Zisser group for all measurements with Wilcoxon signed ranks test revealed no significant difference, except PNS-ANS distance (*P* = 0.02) (Table 2, Figure 3). Comparisons between T1 and T2 within the Le Fort I group for all measurements with the Wilcoxon signed ranks test revealed significant differences concerning PNS-PPW distance, PNS-PPW90 distance, and velopharyngeal area (*P* = 0.03, 0.03, and 0.03, resp.) (Table 2, Figure 4).

Comparisons between T2 and T3 within the Zisser group for all measurements with the Wilcoxon signed ranks test revealed no significant difference, except PNS-U distance (*P* = 0.03) (Table 3, Figure 3). The comparison between T2 and T3 within the Le Fort group for all measurements with the Wilcoxon signed ranks test revealed no significant differences (Table 3, Figure 4).

**Figure 5:**
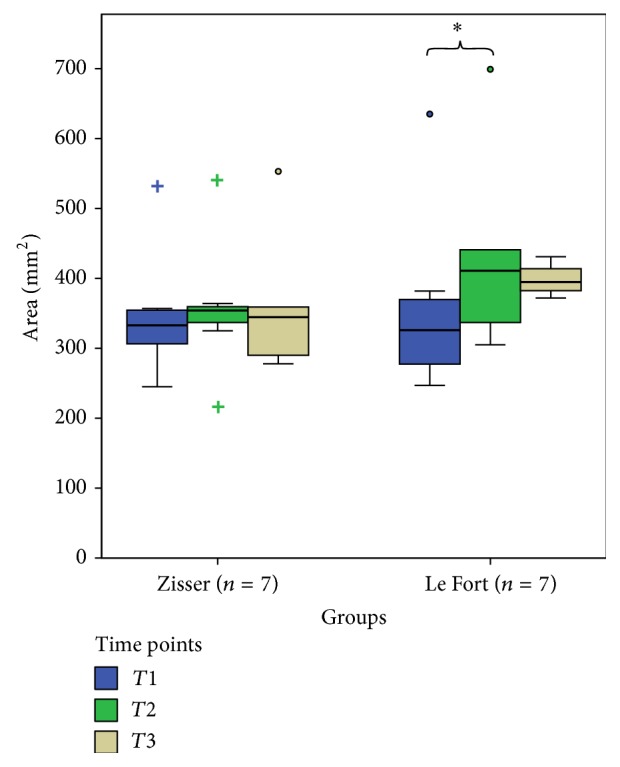
A statistically significant difference was found between T1 and T2 measurements for the area of velopharyngeal space in the Le Fort group. (Box plots show the median, interquartile range, 95% percentile, and outliers as circles and extreme values as plus signs. *∗* indicates significant difference.)

Le Fort I group's patients' velopharyngeal area measurements, however, showed a significant change between T1 and T2 but not between T2 and T3 (*P* = 0.03 and 1.0, resp.) (Figure 5). On the other hand Zisser group's measurements showed no significant change. This supports that conventional Le Fort I osteotomy increases velopharyngeal space and may cause velopharyngeal insufficiency but Zisser's osteotomy does not.

**Figure 6:**
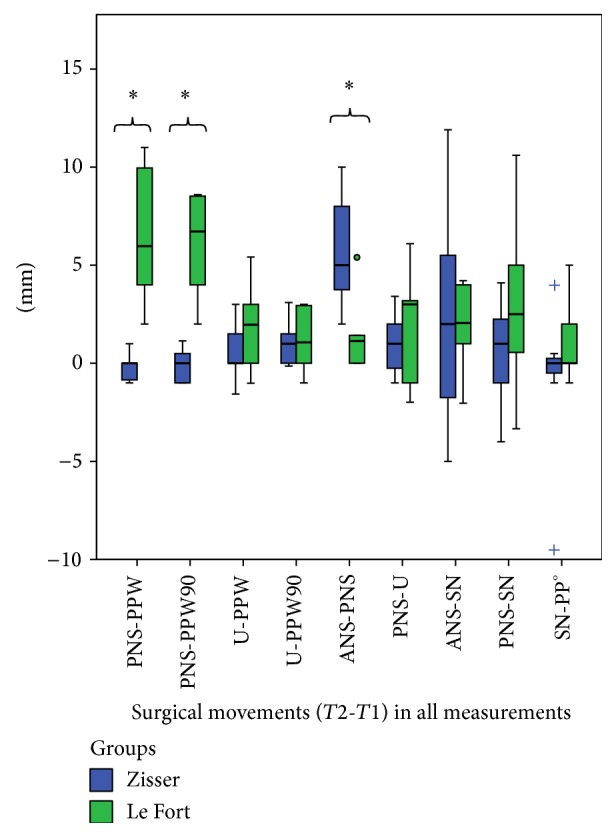
Statistically significant differences were found between Zisser and Le Fort groups' surgical changes (T2-T1) regarding the PNS-PPW, PNS-PPW90, and ANS-PNS distances. (Box plots show the median, interquartile range, 95% percentile, and outliers as circles and extreme values as plus signs. *∗* indicates significant difference.)

The Le Fort I group versus Zisser group relationship regarding the T2-T1 (surgical changes) values with the Mann-Whitney tests revealed no significant differences between the differences at all time points except PNS-PPW, PNS-PPW90, and PNS-ANS distances and the measured area (*P* = 0.003, *P* = 0.003, *P* = 0.02, and *P* = 0.02, resp.) (Figure 6). This also supports that there is a significant difference between Le Fort osteotomy and Zisser osteotomy's sagittal pharyngeal tracings.

**Figure 7:**
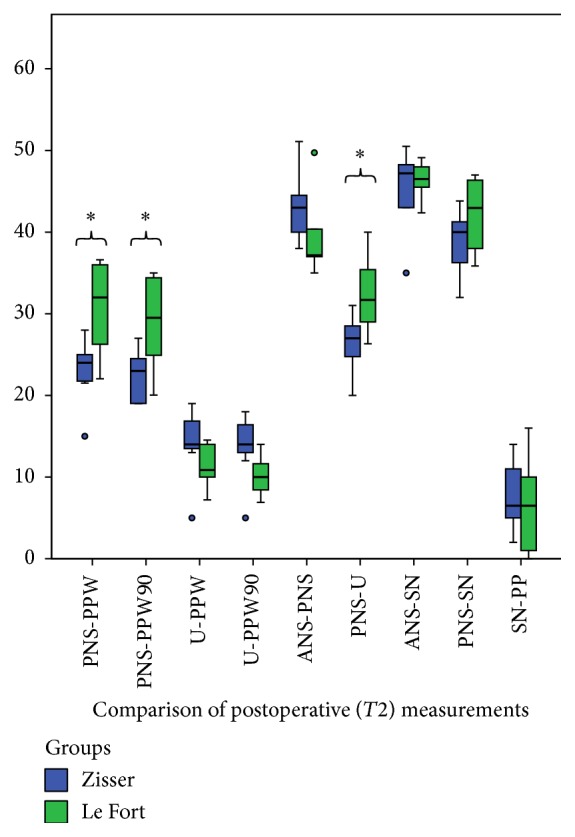
A statistically significant difference was found between the Zisser and Le Fort groups' measurements of T1 regarding the U-PPW distance. (Box plots show the median, interquartile range, 95% percentile, and outliers as circles and extreme values as plus signs. *∗* indicates significant difference.)

**Figure 8:**
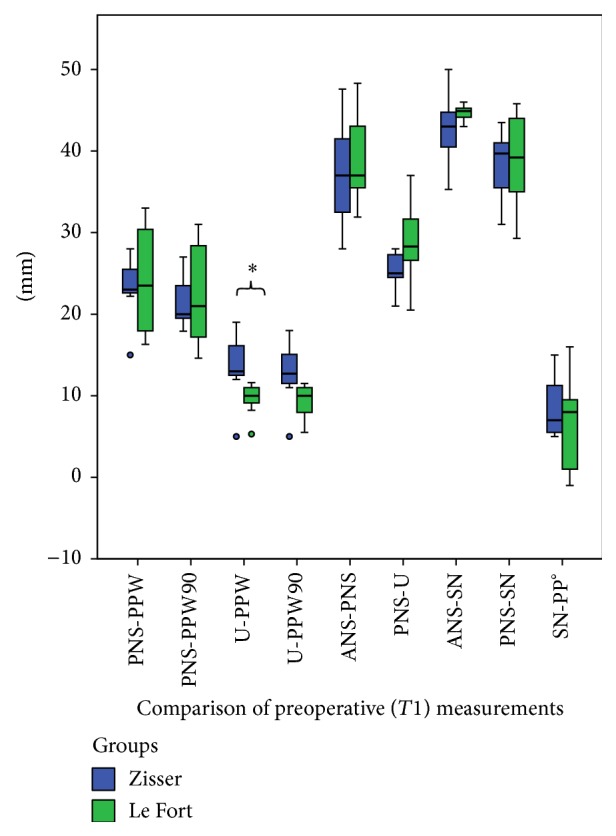
Statistically significant differences were found between the Zisser and Le Fort groups' measurements of T2 regarding the PNS-PPW, PNS-PPW90, and PNS-U distances. (Box plots show the median, interquartile range, 95% percentile, and outliers as circles and extreme values as plus signs. *∗* indicates significant difference.)

The comparison of all measured parameters on T1 time points between the Zisser and Le Fort groups showed a significant difference in the evaluation of the U-PPW distance (*P* = 0.03) (Figure 7). Additionally, the results of the PNS-PPW, PNS-PPW90, and PNS-U distances on the T2 time points between the Zisser and Le Fort groups were significantly different (*P* = 0.02, 0.04, and 0.04, resp.) (Figure 8).

Distraction versus nondistraction comparison with Mann-Whitney tests at the T1 time point revealed no significant difference for all parameters measured at the three time points.

Cleft versus noncleft comparison with Mann-Whitney tests at the T1 time point revealed no significant difference, except the velopharyngeal area of T1 (*P* = 0.04).

There is a negative correlation between the amount of maxillary advancement and area of velopharyngeal space measured at T1 (*P* = 0.004) and cleft palate and area of the velopharyngeal space measured at T1 (*P* = 0.03) for all patients. A positive correlation was found between maxillary advancement and SN-PP angle measured on T2 (*P* = 0.049). Other correlations revealed no significant relationship.

Intraobserver reliability was ≥0.95 for all representative measurements.

## 4. Discussion

Velopharyngeal insufficiency and hypernasality are serious problems commonly observed in cleft lip palate patients. These patients have increased risk of speech deterioration, especially after maxillary advancement procedures [17–19]. Velopharyngeal closure is a complex mechanism affected by multiple factors, such as the soft palate's length, function, and posture, the dimensions of the nasopharynx, and the activity of the posterior and lateral pharyngeal walls, according to Mazaheri et al. [20]. There is no single method to assess all of these factors affecting velopharyngeal function. Static measurement on lateral cephalograms gives information for the morphological changes in the velopharyngeal anatomy [21]. Based on the previous literature related to velopharyngeal evaluation, the cephalometric landmarks used in this study were chosen [21, 22].

Different authors provided clear evidence showing the deleterious effect of maxillary advancement and clearly documented that the forward shift of the maxilla produced velopharyngeal inadequacy and hypernasal resonance [10–12, 23]. There was a significant increase in the measurements of PNS-PPW, U-PPW, and the area of the velopharyngeal space, which may have a potential role in velopharyngeal insufficiency with one-segment maxillary advancement in the current study as well.

Zisser's approach was described especially for retrusive hypoplastic maxilla cases, such as patients with cleft lip palate [14]. Osteotomy between the second premolar and first molar, as well as advancement of the anterior segment, provides maxillary advancement without a deleterious effect on the velopharyngeal space, in theory. The main advantage of the technique is that the position of the soft palate is not changed substantially, and it is expected that speech impediments such as those possibly arising following Le Fort I osteotomy are possibly obviated. Another positive effect of Zisser maxillary advancement is the effective closure of the anterior open bite. However, no study in the current literature revealed any quantitative measurement for the evaluation of Zisser maxillary advancement regarding the velopharyngeal structure and functionality. It is shown that Le Fort group patients show significant increase regarding PNS-PPW and velopharyngeal area on the lateral cephalogram (Table 2, Figure 6). On the other hand, the Zisser group showed no significant change in the mentioned measurements. Therefore, we suggest that maxillary segmental advancement with Zisser osteotomy will not compromise velopharyngeal function.

Some authors claimed that maxillary advancement might improve or worsen certain aspects of speech in patients [18, 24]. Different authors suggest that maxillary advancement may improve articulation due to correction of the occlusion but cause hypernasal speech. It is shown that assessment of palatal length and pharyngeal depth on cephalometric radiographs is helpful in predicting postoperative velopharyngeal insufficiency development [19, 25]. Therefore, we used cephalometric parameters showing pharyngeal depth as a predictor of velopharyngeal insufficiency and compared two maxillary advancement techniques with these parameters.

Zisser osteotomy not only has the advantage of preventing risk of increased velopharyngeal space but also helps to increase the sagittal length of the maxilla, which is important for gaining extra space for prosthodontical treatment in patients with short maxilla. Significant increase in the distance of ANS-PNS with Zisser osteotomy in the current study also showed Zisser osteotomy's effect on maxillary lengthening (Table 2, Figure 6).

One limitation of the present study is the small sample size. Since the indication for Zisser osteotomy is rare, setback and three-piece osteotomies are excluded, and only patients with advancement of more than 4 mm were included in the study. Thus, we had relatively few cases. Another limitation of the study is the lack of functional evaluation, such as video fluoroscopy. However, the main aim of our study was to file the changes of the static cephalometric parameters regarding the morphology of the velopharyngeal structures. It is already shown in the literature that increased velopharyngeal space is associated with increased risk of velopharyngeal insufficiency after the maxillary advancement procedures [12, 19, 23, 26, 27]. The Zisser osteotomy group revealed no significant changes in PNS-PPW and U-PPW distances, whereas the Le Fort I osteotomy group evidenced significant changes in the same distances, including an extension of the upper airways and an increase in the velopharyngeal space.

## 5. Conclusion

Zisser's anterior segmental osteotomy is a reliable procedure for advancement of maxilla with respect to morphological changes in the velopharyngeal structures, especially sagittal measurements and measurements of area of velopharyngeal space on lateral cephalograms. Zisser's osteotomy may become the best solution in selected cases, such as cleft patients who have anterior open bites and increased risk of postoperative velopharyngeal insufficiency.

## Figures and Tables

**Table 1:** Patient characteristics.

Group	Age	Advancement (left/right)	Distraction	Cleft	Follow-up (month)	Mandibular setback
Le Fort	16	11 mm	+	+	21	−
Le Fort	23	5 mm	−	−	1	−
Le Fort	36	7.5 mm/9.5 mm	−	−	11	+
Le Fort	20	9.5 mm	+	−	13	−
Le Fort	15	10 mm	+	+	24	−
Le Fort	17	7.5 mm	+	+	7	−
Le Fort	32	4 mm	−	−		+
Zisser	16	5.5 mm	+	−	1	−
Zisser	21	4 mm	−	+	1	−
Zisser	19	9 mm/7 mm	+	+	62	−
Zisser	17	7 mm	−	+	23	−
Zisser	20	8 mm	−	+	47	−
Zisser	25	4 mm	−	+	1	−
Zisser	16	9.5 mm/10.5 mm	+	+	28	+

**Table 2:** Surgical movements of all points and area for the Zisser and the Le Fort groups. Wilcoxon signed ranks test was used for the comparison.

Surgical movements (T2-T1)	Mean	SD	*P*
PNS-PPW			
Zisser (*N* = 7)	−0.2	0.7	0.45
Le Fort (*N* = 7)	6.5	3.6	0.03^*^
PNS-PPW90			
Zisser (*N* = 7)	−0.1	0.9	0.52
Le Fort (*N* = 7)	6.1	2.7	0.03^*^
U-PPW			
Zisser (*N* = 7)	0.6	1.5	0.27
Le Fort (*N* = 7)	1.9	2.4	0.14
U-PPW90			
Zisser (*N* = 7)	1.0	1.2	0.08
Le Fort (*N* = 7)	1.2	1.7	0.14
PNS-ANS			
Zisser (*N* = 7)	5.8	2.9	0.02^*^
Le Fort (*N* = 7)	1.5	2.0	0.07
PNS-U			
Zisser (*N* = 7)	1.0	1.6	0.14
Le Fort (*N* = 7)	2.1	3.0	0.12
SN-ANS90			
Zisser (*N* = 7)	2.3	5.9	0.35
Le Fort (*N* = 7)	1.9	2.3	0.12
SN-PNS90			
Zisser (*N* = 7)	0.5	2.7	0.50
Le Fort (*N* = 7)	3.0	4.8	0.12
SN-PP angle			
Zisser (*N* = 7)	−0.9	4.1	0.72
Le Fort (*N* = 7)	1.0	2.2	0.29
Area			
Zisser (*N* = 7)	10.4	21.9	0.18
Le Fort (*N* = 7)	66.4	62.2	0.03^*^

^*∗*^Significant difference.

**Table 3:** Postsurgical movements of all points and area for the Zisser and the Le Fort groups. Wilcoxon signed ranks test was used for the comparison.

Postsurgical movements (T3-T2)	Mean	SD	*P*
PNS-PPW			
Zisser (*N* = 7)	2.0	5.4	1.0
Le Fort (*N* = 7)	−1.6	3.9	0.59
PNS-PPW90			
Zisser (*N* = 7)	2.7	3.7	0.69
Le Fort (*N* = 7)	−1.7	3.0	0.29
U-PPW			
Zisser (*N* = 7)	−1.0	2.1	0.30
Le Fort (*N* = 7)	−0.3	2.1	0.59
U-PPW90			
Zisser (*N* = 7)	−0.9	22	0.60
Le Fort (*N* = 7)	0.3	1.5	1.0
PNS-ANS			
Zisser (*N* = 7)	−0.2	0.8	0.41
Le Fort (*N* = 7)	−0.3	7.5	0.66
PNS-U			
Zisser (*N* = 7)	−2.7	1.8	0.03^*^
Le Fort (*N* = 7)	−1.6	2.1	0.29
SN-ANS90			
Zisser (*N* = 7)	0.3	3.1	0.89
Le Fort (*N* = 7)	2.8	1.4	0.11
SN-PNS90			
Zisser (*N* = 7)	0.9	2.2	0.35
Le Fort (*N* = 7)	2.4	2.0	0.11
SN-PP angle			
Zisser (*N* = 7)	−0.3	1.4	0.58
Le Fort (*N* = 7)	−3.0	3.6	0.18
T3.area			
Zisser (*N* = 7)	3.0	32.7	0.92
Le Fort (*N* = 7)	1.0	52.4	1.0

^*∗*^Significant difference.

## References

[1] Li Y., Shi B., Song Q.-G., Zuo H., Zheng Q. (2006). Effects of lip repair on maxillary growth and facial soft tissue development in patients with a complete unilateral cleft of lip, alveolus and palate. *Journal of Cranio-Maxillofacial Surgery*.

[2] Ross R. B. (1987). Treatment variables affecting facial growth in complete unilateral cleft lip and palate. *Cleft Palate Journal*.

[3] Lu Y., Shi B., Zheng Q., Xiao W., Li S. (2006). Analysis of velopharyngeal morphology in adults with velopharyngeal incompetence after surgery of a cleft palate. *Annals of Plastic Surgery*.

[4] Reyneke J. P., Reyneke J. P. (2003). Principles of orthognathic surgery. *Essentials of Orhognathic Surgery*.

[5] Haapanen M.-L., Kalland M., Heliövaara A., Hukki J., Ranta R. (1997). Velopharyngeal function in cleft patients undergoing maxillary advancement. *Folia Phoniatrica et Logopaedica*.

[6] Johanson B., Ohlsson A., Friede H., Ahlgren J. (1974). A follow up study of cleft lip and palate patients treated with orthodontics, secondary bone grafting, and prosthetic rehabilitation. *Scandinavian Journal of Plastic and Reconstructive Surgery*.

[7] Gaggl A., Schultes G., Kärcher H. (1999). Aesthetic and functional outcome of surgical and orthodontic correction of bilateral clefts of lip, palate, and alveolus. *The Cleft Palate-Craniofacial Journal*.

[8] Keller B. G., Long R. E., Gold E. D., Roth M. D. (1988). Maxillary dental arch dimensions following pharyngeal-flap surgery. *Cleft Palate Journal*.

[9] Good P. M., Mulliken J. B., Padwa B. L. (2007). Frequency of Le Fort I osteotomy after repaired cleft lip and palate or cleft palate. *Cleft Palate-Craniofacial Journal*.

[10] Scheuerle J. (1999). Commentary on velopharyngeal changes after maxillary advancement in cleft patients with distraction osteogenesis using a rigid external distraction device: a 1-year cephalometric follow-up. *Journal of Craniofacial Surgery*.

[11] Okazaki K., Satoh K., Kato M., Iwanami M., Ohokubo F., Kobayashi K. (1993). Speech and velopharyngeal function following maxillary advancement in patients with cleft lip and palate. *Annals of Plastic Surgery*.

[12] Watzke I., Turvey T. A., Warren D. W., Dalston R. (1990). Alterations in velopharyngeal function after maxillary advancement in cleft palate patients. *Journal of Oral and Maxillofacial Surgery*.

[13] Zisser G. (1972). Surgical correction of alveolar malposition. *Deutsche Zahn-, Mund-, und Kieferheilkunde mit Zentralblatt für die Gesamte*.

[14] Zisser G. (1969). Surgical treatment of maxillary retrusion. *Zahnarztliche Praxis*.

[15] James D. R., Brook K. (1985). Maxillary hypoplasia in patients with cleft lip and palate deformity—the alternative surgical approach. *The European Journal of Orthodontics*.

[16] Sell D., Ma L., James D., Mars M., Sheriff M. (2002). A pilot study of the effects of transpalatal maxillary advancement on velopharyngeal closure in cleft palate patients. *Journal of Cranio-Maxillofacial Surgery*.

[17] Cheung L. K., Chua H. D. P., Hägg M. B. (2006). Cleft maxillary distraction versus orthognathic surgery: clinical morbidities and surgical relapse. *Plastic and Reconstructive Surgery*.

[18] Janulewicz J., Costello B. J., Buckley M. J., Ford M. D., Close J., Gassner R. (2004). The effects of Le Fort I osteotomies on velopharyngeal and speech functions in cleft patients. *Journal of Oral and Maxillofacial Surgery*.

[19] McComb R. W., Marrinan E. M., Nuss R. C., Labrie R. A., Mulliken J. B., Padwa B. L. (2011). Predictors of velopharyngeal insufficiency after le Fort I maxillary advancement in patients with cleft palate. *Journal of Oral and Maxillofacial Surgery*.

[20] Mazaheri M., Athanasiou A. E., Long R. E. (1994). Comparison of velopharyngeal growth patterns between cleft lip and/or palate patients requiring or not requiring pharyngeal flap surgery. *The Cleft Palate-Craniofacial Journal*.

[21] Satoh K., Wada T., Tachimura T., Shiba R. (2002). The effect of growth of nasopharyngeal structures in velopharyngeal closure in patients with repaired cleft palate and controls without clefts: a cephalometric study. *British Journal of Oral and Maxillofacial Surgery*.

[22] Yu H., Wang X., Fang B., Shen S. G. (2012). Comparative study of different osteotomy modalities in maxillary distraction osteogenesis for cleft lip and palate. *Journal of Oral and Maxillofacial Surgery*.

[23] Witzel M. A., Munro I. R. (1977). Velopharyngeal insufficiency after maxillary advancement. *Cleft Palate Journal*.

[24] Voshol I. E., Van Der Wal K. G. H., Van Adrichem L. N. A., Ongkosuwito E. M., Koudstaal M. J. (2012). The frequency of Le Fort I osteotomy in cleft patients. *Cleft Palate-Craniofacial Journal*.

[25] D'Antonio L. L., Eichenberg B. J., Zimmerman G. J. (2000). Radiographic and aerodynamic measures of velopharyngeal anatomy and function following Furlow Z-plasty. *Plastic and Reconstructive Surgery*.

[26] Kummer A. W., Strife J. L., Grau W. H., Creaghead N. A., Lee L. (1989). The effects of Le Fort I osteotomy with maxillary movement on articulation, resonance, and velopharyngeal function. *Cleft Palate Journal*.

[27] Schendel S. A., Oeschlaeger M., Wolford L. M., Epker B. N. (1979). Velopharyngeal anatomy and maxillary advancement. *Journal of Maxillofacial Surgery*.

